# Multilocus evidence provides insight into the demographic history and asymmetrical gene flow between *Ostrinia furnacalis* and *Ostrinia nubilalis* (Lepidoptera: Crambidae) in the Yili area, Xinjiang, China

**DOI:** 10.1002/ece3.9504

**Published:** 2022-11-16

**Authors:** Bing Li, Zhaofu Yang

**Affiliations:** ^1^ College of Plant Protection Northwest A&F University Yangling China; ^2^ Key Laboratory of Plant Protection Resources and Pest Mangagement Northwest A&F University Yangling China

**Keywords:** Crambidae, gene flow, genetic differentiation, geographic isolation, *Ostrinia*

## Abstract

1. Tianshan Mountains provide a model for studying biological evolution and speciation. Here we assess the evolutionary history of *Ostrinia furnacalis* (ACB) and *Ostrinia nubilalis* (ECB), which are sympatric in the Yili River Valley in Xinjiang, China.
2. Our study is based on the historical gene flow analyses of two species by using three mitochondrial DNA (mtDNA, *COI*, *COII*, *Cytb*) and four nuclear DNA (nuDNA, *EF‐1α*, *Wingless*, *RPS5*, *CAD*) markers obtained from representatives of HC (Huocheng), YN (Yining), XY (Xinyuan), and MNS (Manasi).
3. Our results reveal that there is an asymmetrical gene flow pattern between the four populations. The population migratory pathways between these different populations show inflow into HC and YN, outflow from XY, and that MNS maintained a flow balance. Bayesian divergence time dating based on the *COI* gene suggests that the genetic divergence between the two species in this area may have occurred in Holocene at 0.008 Mya. Neutrality tests (Tajima's *D*, Fu's *F*_s_), and mismatch distribution test results suggest that population expansion events may not have occurred in the recent past. The demographic history and gene flow pattern between ACB and ECB may follow the “mountain isolation” hypothesis. The ML and BI trees of the mtDNA haplotype dataset show that ECB haplotypes are grouped together in a distinct clade and are clearly separate from ACB haplotypes. However, the geographical pattern of haplotype distribution is less clear for both ACB and ECB, supporting that there has been frequent gene flow among the geographic populations in the Tianshan Mountains.
4. These findings indicate that the Tianshan Mountains are less likely a barrier to gene flow of the two species.

## Graphical abstract

Tianshan Mountain provides a model for studying biological evolution and speciation. Here we assess the evolutionary history of the *Ostrinia furnacalis* and *Ostrinia nubilalis*, which are sympatric in the Yili River Valley in Xinjiang, China. Our study is based on the historical gene flow analyses of two species by using three mitochondrial DNA (mtDNA, *COI*, *COII*, *Cytb*) and four nuclear DNA (nuDNA, *EF‐1α*, *Wingless*, *RPS5*, *CAD*) markers obtained from representatives of HC (Huocheng), YN (Yining), XY (Xinyuan) and MNS (Manasi).

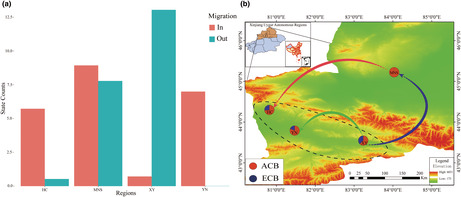

## 1 INTRODUCTION

Deciphering genetic variations and demographic history of the existing distribution patterns of different populations within and between species have been a major focus in biogeography for decades (Avise, 1992; Yao et al., 2021). Several biogeographic hypotheses asserting that diversification and speciation were driven by isolation of different populations, e.g., “glacial refugia” hypothesis (Avise, 1992; Smith, 1965) and “mountain isolation” hypothesis (Avise, 1998). These hypotheses assume that the different populations were partially or completely isolated by glaciers or mountains during the glacial period, and the genetic differences of isolated populations gradually accumulated with genetic drift and/or natural selection (Willis et al., 2004). Therefore, the complex historical trajectories linked to species divergence have posited to understand current population genetic processes and to predict the potential for populations to respond to selection and divergent environmental conditions. However, the study that focuses on testing biogeographic hypotheses is scarce in biodiversity hotspots in Central Asia, particularly in the northwestern of China (Yao et al., 2021).

The Tianshan Mountains of Xinjiang experienced complex orogenic and climatic events during the Pleistocene (0.0117–2.588 Mya) and the Holocene (present to 0.0117 Mya) are located in the northwestern China and provide an excellent model for inferring biogeographical processes associated with species divergence according to proposed biogeographic hypotheses (Sun et al., 2004). The topography and landforms of the Tianshan Mountains became very complicated, ranging from 300 to 7500 m above sea level (Hewitt, 1996) and were heavily influenced by the uplift of the Qinghai–Tibet Plateau. A series of large and small mountain ranges are running north–south and form the North, Middle, and South Tianshan (e.g., Alatau Mountain, Boluokenu Mountain, Narathi Mountain) with a number of intermountain basins (e.g., Junggar Basin, Yili Basin, Tarim Basin) and the Yili River Valley. Many glacial refuges are found within these complex landforms (Médail & Diadema, 2009), which are considered as important glacial refuge hotspots. Some species may have experienced frequent distribution expansion and contraction during glacial–interglacial cycles, leading to potential gene flow between isolated populations in refugia on the Tianshan Mountains (Brower & Desalle, 1998; Qu et al., 2011, 2014; Song et al., 2009). However, the Tianshan Mountains may act as a barrier to gene flow between geographically isolated populations and close relatives, resulting in spatial genetic differentiation and the current complex population structure of a sibling species complex. For example, the Qinghai–Tibet Plateau, the south of the Tianshan Mountains, and its surrounding regions contributed to the divergence between *Primula nutans* and *Primula fasciculata* (Ren et al., 2018).

**FIGURE 1:**
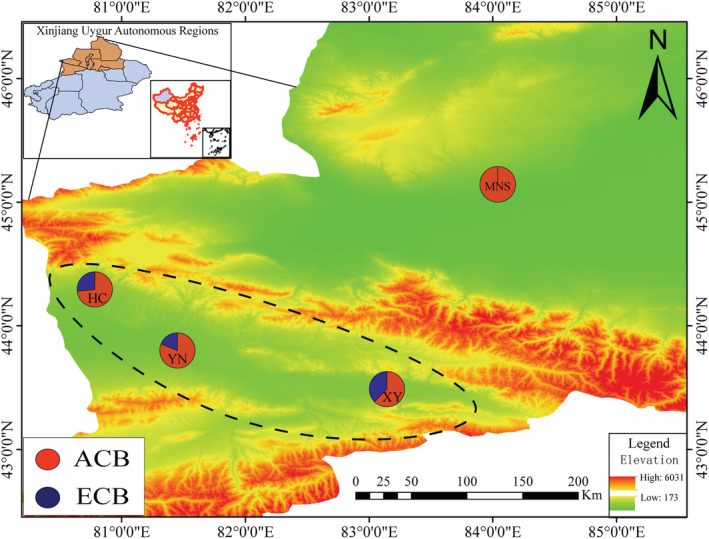
Topographical map of Central Xinjiang, showing sampling sites and genetic lineages within *Ostrinia furnacalis* (ACB) and *Ostrinia nubilalis* (ECB). Circles represent sampling sites (YN, XY, HC and MNS) and are proportional to the sample size of two species. The black dashed line represents the approximate border of the Yili River valley of the Tianshan Mountains. Detailed sampling information is shown in Appendix S1.

The ACB (Asian corn borer), *O. furnacalis* (Guenée), and the ECB (European corn borer), *O. nubilalis* (Hübner) (Crambidae: Pyraustinae), are worldwide maize pests that cause substantial yield losses in corn production (Bourguet et al., 2014; Frolov et al., 2007; Mutuura & Munroe, 1970). These two corn borers both belong to the *O. nubilalis* species group (trilobed uncus of male genitalia, which is a structure derived from the 10th abdominal tergite to grasp the female during copulation; see Yang et al., 2021: 830, Clade III in Figure 1), one of the most evolutionarily and ecologically interesting but taxonomically difficult groups in Lepidoptera. The species group includes 10 species and 23 subspecies worldwide (Frolov et al., 2007; Mutuura & Munroe, 1970; Yang et al., 2021). Incongruence between molecular phylogenetic relationships and the traditional classification of *Ostrinia* has been puzzling for a long time, leading to a number of members including ACB and ECB being morphologically indistinguishable and making accurate species identification extremely difficult (Hoshizaki et al., 2008; Kim et al., 1999; Mutuura & Munroe, 1970; Wang et al., 2017; Yang et al., 2021). On the other hand, this species complex is a good example to understand biogeographic patterns and evolutionary histories owing to their distributional complexity of the world. In these species, the ECB has spread from Europe to the central regions of Asia, as far east as Uzbekistan and to the western edge of Xinjiang (Xinjiang Uyghur Autonomous Region) in China, while the ACB has been reported to damage maize in eastern Asia, Northern Australia (Nafus & Schreiner, 1991), and in the major maize‐growing regions of Eastern China (Hu & Sun, 1979; Tang et al., 1988).

Early studies indicated that only ECB occurred in the Yili area of the Western Xinjiang (Li et al., 1982; Huang et al., 2017; Tang et al., 1988). Recent studies now indicate that ACB also occurs in this area (Li et al., 2010, 2013, 2014; Yang et al., 2008, 2011). There are now some regions in the Yili Kazak Autonomous Prefecture Area of Xinjiang where ACB and ECB co‐occur (Wang et al., 2017). As a result, it is the only known area in the world where these two corn borer species, ACB and ECB, have made contact. This provides the rare opportunity to study the evolution of sympatric sibling species in situ (Wang et al., 2017). Previous studies on the genus *Ostrinia* showed that assortative mating is evident among sympatric ECB and *Ostrinia scapulalis* (the Adzuki bean borer) in Europe based on pheromone race‐specific gene marker genetic analysis (Malausa et al., 2005). Coates et al. (2018) utilized a single‐nucleotide polymorphism (SNP) at the *pgfar* locus to study the genomic mechanisms of sympatric ecological and sexual divergence of ECB, illuminating the genetic basis of strain‐specific adaptive traits and gene flow between ECB strains. Wang et al. (2017) proposed that there was introgression of genes and the presence of hybrid individuals between ACB and ECB within the Yili area, suggesting that incomplete lineage sorting and historical gene flow may shape the evolutionary trajectory of these two species in this area.

However, any assumption about biogeographic patterns of genetic variation and gene flow patterns within and between *Ostrinia* species, including the migratory pathways of possible gene flow and potential barrier to gene flow among different populations in sympatric areas of the Yili area and in allopatric regions isolated by the Tianshan Mountains, remains largely unknown. In this study, we investigated the gene flow patterns of ACB and ECB by reconstructing population evolutionary history of each species. The primary goals were (i) to reveal gene flow direction of ACB and ECB in sympatric regions within the Yili area and on both sides of the Tianshan Mountains; (ii) to determine the demographic history and diffusion pathways between the different populations of ACB and ECB; and (iii) to explore whether any possible barriers in the Tianshan Mountains are expected to impact their gene flow and biogeographical patterns on both sides, which may follow the “mountain isolation” hypothesis.

## 2 MATERIALS AND METHODS

### 2.1 Sampling and laboratory procedures

Adult specimens were collected from four regions in Xinjiang, China during July and August 2017, including three regions in the Yili Kazak Autonomous Prefecture Area where ACB and ECB co‐occur. We defined these four regions, namely Huocheng, Yining, Xinyuan, and Manasi, as the four geographical populations HC, YN, XY, and MNS, respectively. The former three populations, namely HC, YN, XY, are located in the Yili River Valley, and MNS population is situated on the other side of the Tianshan Mountains. Specimens were collected using both sweeping net and light trap methods and have been stored as pinned dry collection at the Entomological Museum, Northwest A&F University, Yangling, Shaanxi Province, China (NWAFU). Some of the adult specimens were preserved in 95% ethanol at −20°C at the NWAFU. We selected 15–19 samples from each population for DNA extraction (Appendix S1).

### 2.2 DNA extraction, PCR, and sequencing

Genomic DNA was extracted from one or two legs of adult specimens using the DNeasy DNA Extraction Kit (TransGen Biotech) following the manufacturer's protocols. Three mitochondrial and four nuclear gene markers were obtained including: *COI* (cytochrome coxidase subunit I), *COII* (cytochrome coxidase subunit II), *Cytb* (cytochrome b), *EF‐1α* (elongation factor‐1α), *Wingless* (wingless), *RPS5* (ribosomal protein S5), and *CAD* (carbamoyl phosphate synthetase/aspartate transcarbamylase/dihydroorotase). PCR primers used in this study are listed in Appendix S2. The PCR amplifications were conducted in a total volume of 25 μl comprising: 12.5 μl PCR MasterMix, 8.5 μl molecular grade water, 1 μl forward and reverse primers (10 μM), and 2 μl DNA template (100 ng/μl). The PCR profile comprised an initial denaturation for 3 min at 95°C; 37 cycles consisting of denaturing for 45 s at 94°C; followed by annealing for 1 min at 51°C (*COI*), 52°C (*COII*), 45 °C (*Cytb*), 57°C (*EF‐1α*), 60°C (*Wingless*), 58°C (*RPS5*), 45°C (*CAD*), and an extension for 1 min at 72°C; a final extension step of 72°C for 10 min. PCR products were detected by 1.0% agarose gel electrophoresis, stained with ethidium bromide, and visualized by ultraviolet light. DNA products were subsequently bidirectionally sequenced by AuGCT Biotech.

Sequence chromatograms were checked by using Chromas Pro v 2.6.5 (Technelysium Pty Ltd.) and edited through MAFFT (Katoh et al., 2002) in Geneious 8.1.3 (Biomatters) and BioEdit v 7.0.9.0 (Hall, 1999). The identification of specimens used for molecular analyses was verified through a GenBank BLAST search, the Barcode of Life Database (BOLD; Ratnasingham & Hebert, 2007), and genitalia examination (Yang et al., 2021), namely 12 of ACB and 6 of ECB in XY, 12 of ACB and 6 of ECB in YN, 11 of ACB and 4 of ECB in HC, 19 of ACB in MNS, respectively (Figure 1, Appendix S1). Voucher specimens and corresponding mounted slides were deposited at the NWAFU. All newly generated sequences have been submitted to GenBank with the following accession numbers: *COI*: MK286109‐MK286173; *COII*: MK286174‐MK286237; *Cytb*: MK286307‐MK286374; *EF‐1α*: MK303832‐MK303897; *Wingless*: MK292217‐MK292285; *RpS5*: MK286238‐MK286306; *CAD*: MK292148‐MK292216.

### 2.3 Genetic polymorphism and population structure analyses

To evaluate the variation in genetic diversity among the four sampled geographical populations, we calculated the number of haplotypes (*N*_h_), haplotype diversity (*H*_d_), and nucleotide diversity (*π*) for each population of different species using DnaSP v 6.12.03 (Rozas & Librado, 2017) based on the datasets of the combined three mitochondrial genes (MTD) and the combined four nuclear genes (NUD), respectively. The mtDNA haplotype dataset (MTHD) and nuDNA haplotype dataset (NUHD) were defined separately using DnaSP v 6.12.03 (Rozas & Librado, 2017) based on the MTD and NUD. The defined MTHD and NUHD were used in the subsequent phylogenetic analyses. The haplotype networks of the two species were constructed using TCS (Templeton, Crandall, and Sing's parsimonious network, Templeton et al., 1992) network strategies in PopART (Bandelt et al., 1999; Clement et al., 2002) based on MTHD.

Genetic distances within and between populations were calculated in MEGA v 11. 08 (Tamura et al., 2021) employing the Kimura 2‐parameter (K2P) model (Kimura, 1980; Kumar et al., 2016). To evaluate patterns of isolation by distance, we tested the correlation between genetic distances and geographical distances among populations by using a Mantel test in NTSYSpc‐2.10 e with 1000 permutations (Peakall & Smouse, 2006; Rohlf, 1997).

An analysis of molecular variance (AMOVA) was used to investigate the genetic variation between and within populations in the Arlequin 3.5 software (Excoffier & Lischer, 2010) with 1000 permutations based on MTD and NUD, respectively.

Genetic differentiation (*F*_st_) of different geographical populations for each species was calculated in the DnaSP v 6.12.03 software (Rozas & Librado, 2017) based MTHD and NUHD, respectively. The levels of genetic differentiation were determined as *F*_st_ < 0.05 (negligible differentiation), 0.05 < *F*_st_ < 0.15 (moderate differentiation), 0.15 < *F*_st_ < 0.25 (relatively large differentiation), and *F*_st_ > 0.25 (great differentiation; Rousset, 1997; Wright, 1978).

### 2.4 Phylogenetic analyses

A sequence saturation test was carried out for MTHD and NUHD, respectively, by using the DAMBE software (Xia & Lemey, 2009). The phylogenetic relationships of different haplotypes were reconstructed with maximum likelihood (ML) and Bayesian inference (BI) based on MTHD and NUHD. Both ML and BI were used for each dataset. The species *Ostrinia latipennis* (Warren) (H27) was selected as the outgroup in the phylogenetic analyses. ML and BI analyses were executed in the PhyloSuite v 1.2.2 software (Zhang et al., 2020). ML analyses were conducted with 1000 bootstraps and were run 10 times starting from random seeds under the GTRGAMMA model. The best partition schemes and substitution models for each gene of BI analyses were estimated for the MTHD and NUHD using PartitionFinder v 2.1.1 (Lanfear et al., 2017) under Bayesian Information Criterion (BIC). Two independent parallel runs of four incrementally heated Markov Chain Monte Carlo (MCMC) chains (one cold chain and three hot chains) were run for 30 million generations, with trees sampled every 100 generations. The average standard deviation of split frequencies and Potential Scale Reduction Factor (PSRF) were used for examining convergence. Stationarity was determined using Tracer v 1.7.2 (Rambaut et al., 2018) by plotting the log‐likelihood values versus the generation number. The first 25% of the total tree samples were discarded, and the remaining samples were used to generate a majority rule consensus tree and to calculate the posterior probabilities. FigTree v 1.4.3 (Rambaut et al., 2018) was used to visualize the results.

### 2.5 Historical demography and divergence dating analyses

The historical demographic dynamics of different geographical populations for each species was estimated via the effective population size using multiple approaches based on MTD and NUD, respectively. First, Tajima's *D* (Tajima, 1989) and Fu's *F*_s_ (Fu, 1997) statistics were used to assess whether the nucleotide polymorphisms deviated from expectations under the neutral theory with 10,000 coalescent simulations in the Arlequin 3.5 software (Excoffier & Lischer, 2010). Second, mismatch distribution analyses were performed under a model of sudden demographical expansion with 1000 permutations to find any evidence of past demographic expansions using Arlequin 3.5 (Excoffier & Lischer, 2010).

The timing of divergence was estimated in the Beast v 1.10.4 software (Drummond et al., 2018) based on the mitochondrial *COI* marker. We tried the strict molecular clock model and the uncorrelated lognormal relaxed molecular clock model, and compared the Bayes factor model with the Akaike information criterion (AIC) in Tracer v 1.7.2 (Rambaut et al., 2018). The nucleotide substitution model of *COI* marker was selected through MrModeltest v 2.3 (Nylander, 2004) as the “HKY + I” model. Due to the lack of dated fossils or geological events useful for molecular clock dating, we used the widely accepted mutation rates for the insect mitochondrial *COI* marker (0.0177 site^−1^ Ma^−1^; Papadopoulou et al., 2010). Samples were taken every 40,000 generations, and 400 million generations were performed, with the first 10% of samples discarded as burn‐in. Tracer v 1.7.2 (Rambaut et al., 2018) was used to monitor the stability of the chain and the convergence of the two parallel runs to determine whether the effective sample size (ESS) of each parameter reached the recommended value of >200. TreeAnnotator (Drummond et al., 2018) was used to aggregate information from a single post‐burn‐in tree into a single Maximum Cladistic Confidence (MCC) tree, discarding the first 5000 trees as burn‐in trees. FigTree 1.4.3 (Rambaut et al., 2018) was used to visualize the MCC tree.

### 2.6 Gene flow analyses

Relative gene flow (*N*_m_) between geographical populations of the two species and within each species was calculated in the DnaSP v 6.12.03 software (Rozas & Librado, 2017) based on MTD and NUD, respectively. The levels of gene flow were estimated as *N*_m_ < 0.25 (low frequency), 0.25 < *N*_m_ < 0.99 (Moderate frequency), *N*_m_ > 1 (high frequency) (Govindajuru, 1989). In order to investigate the gene flow migratory pathways, we used Bayesian stochastic search variable selection (BSSVS) analyses (Su et al., 2015) based on the MTD. BSSVS analyses were conducted in the BEAST v 1.10.4 software (Drummond et al., 2018) by using the Markov modules of jumps to construct the matrix module. SpreaD 3 (Bielejec et al., 2016) was used to estimate the Bayes Factor (BF) and Indicator between different populations (at a minimum, a migratory pathways with BF values >3 and Indicator values >0.5 was considered as there are potential migratory pathways among the different geographical populations) (Bielejec et al., 2016).

## 3 RESULTS

### 3.1 Genetic polymorphism and genetic diversity

Variation of the seven gene markers of ACB and ECB was shown in Appendix S3. In the concatenated mtDNA alignment, 92 nucleotide positions were variable and 76 were parsimony informative. The analyses of nucleotide composition showed a high A + T content (74.1%). The estimated ratio of transition to transversion was (*R*) = 2.4. In the concatenated nuDNA alignment, 80 nucleotide positions were variable sites, and 46 were parsimony informative. The analysis of nucleotide composition showed a low A + T content (47.5%). The estimated ratio of transition to transversion was (*R*) = 3.8.

Genetic diversity indices were summarized separately for each population and species (Appendix S4). For ACB, haplotype diversity (*H*_d_) of the four geographical populations (YN, XY, HC, MNS) ranged from 0.923 to 1.000 based on MTD while the values of *H*_d_ were ca. 1.000 based on NUD. The nucleotide diversity (*π*) ranged from 0.01046 to 0.01810 and from 0.00347 to 0.00451 based on MTD and NUD, respectively. For ECB, haplotype diversity (*H*_d_) of the three geographical populations (YN, XY, HC) ranged from 0.345 to 0.733 based on MTD while the values of *H*_d_ were ca. 1.000 based on NUD. The nucleotide diversity (*π*) ranged from 0.00030 to 0.00095 and from 0.00357 to 0.00535 based on MTD and NUD, respectively. In general, the total sampling individuals of ACB (0.934 and 0.01366) exhibited a higher *H*_d_ and *π* values than ECB (0.505 and 0.00051) based on MTD, while ACB (1.000 and 0.0041) and ECB (1.000 and 0.00492) showed an approximate *H*_d_ and *π* values based on NUD.

The AMOVA analyses revealed a different pattern of genetic variation based on MTD and NUD. The total variation based on MTD was mainly explained by groups of both species (55.83% of the variation) and within populations (45.65% of the variation), while the total variation based on NUD was mainly accounted for by within populations (73.97% of the variation), compared with that among groups (25.60%) or among populations within groups (0.43%; Appendix S5). Furthermore, the *F*_st_ was applied to detect the genetic differentiation within the two species (ACB and ECB) across their different geographical populations (YN, XY, HC, MNS). Our results showed that the *F*_st_ values between the ACB and ECB ranged from 0.58109 to 0.66714 and from 0.19175 to 0.37037 based on MTD and NUD, respectively. Moreover, for the three sympatric regions (YN, XY, HC), the values of *F*_st_ between the ACB and ECB ranged from 0.58109 to 0.66139 and from 0.22095 to 0.33358 based on MTD and NUD, respectively (Table 1). For ACB, the *F*_st_ values between different geographical populations ranged from −0.11031 to 0.10331 and from −0.0226 to 0.07464 based on MTD and NUD, respectively. For ECB, the *F*_st_ values between different geographical populations ranged from 0 to 0.1 and from −0.06 to 0.0566 based on MTD and NUD, respectively. Our results indicated that the degree of genetic differentiation between the two species (ACB and ECB) was high; however, genetic differentiation among different geographical populations was low for both ACB and ECB.

**TABLE 1:** The genetic differentiation (*F*_st_) and gene flow (*N*_m_) of the two species across their different distribution range based on mitochondrial genes dataset (MTD) and nuclear genes dataset (NUD).

*Ostrinia furnacalis* (ACB)	*Ostrinia nubilalis* (ECB)	*F*_st_	*N*_m_
XY	XY	0.58109/0.22095	0.18/0.44
YN	YN	0.66139/0.33358	0.13/0.25
HC	HC	0.6321/0.30313	0.145/0.575
YN	XY	0.64987/0.19175	0.135/1.054
HC	XY	0.62109/0.23913	0.153/0.8
MNS	XY	0.60826/0.19589	0.161/1.03
XY	YN	0.58909/0.26568	0.17/0.69
XY	HC	0.59492/0.23572	0.17/0.81
HC	YN	0.62731/0.37037	0.15/0.43
MNS	YN	0.61634/0.27869	0.156/0.647
YN	HC	0.66714/0.28687	0.125/0.621
MNS	HC	0.62208/0.23925	0.15/0.79
*O. furnacalis* (ACB)			
XY	YN	−0.01176/0.07464	−21.5/3.1
XY	MNS	−0.06995/−0.01465	−3.82/−17.31
YN	HC	0.10331/−0.01343	2.17/−18.87
YN	MNS	−0.00258/0.01781	−97.15/13.79
HC	MNS	−0.06193/−0.01246	−4.29/−20.31
XY	HC	−0.11031/−0.02261	−2.5/−11.3
*O. nubilalis* (ECB)			
XY	YN	0.1/0.0566	2.25/4.17
XY	HC	0.01053/−0.00629	23.49/−40
YN	HC	0/−0.06	0/−4.42

*Note*: Values reported as follow: MTD/NUD.

The mean genetic distance (K2P) values between different geographical populations ranged from 0.014 to 0.019 and from 0.004 to 0.006 based on MTD and NUD, respectively (Appendix S6). The correlation coefficients between genetic distances and geographical distances were *R* = .39723 (*p* = .8098 > .05) for MTD and *R* = −.23 (*p* = .2986 > .05) for NUD, respectively. These results indicated that there was no significant correlation between genetic differences and geographical distances in either dataset.

### 3.2 Population structure and phylogenetic analyses

**FIGURE 2:**
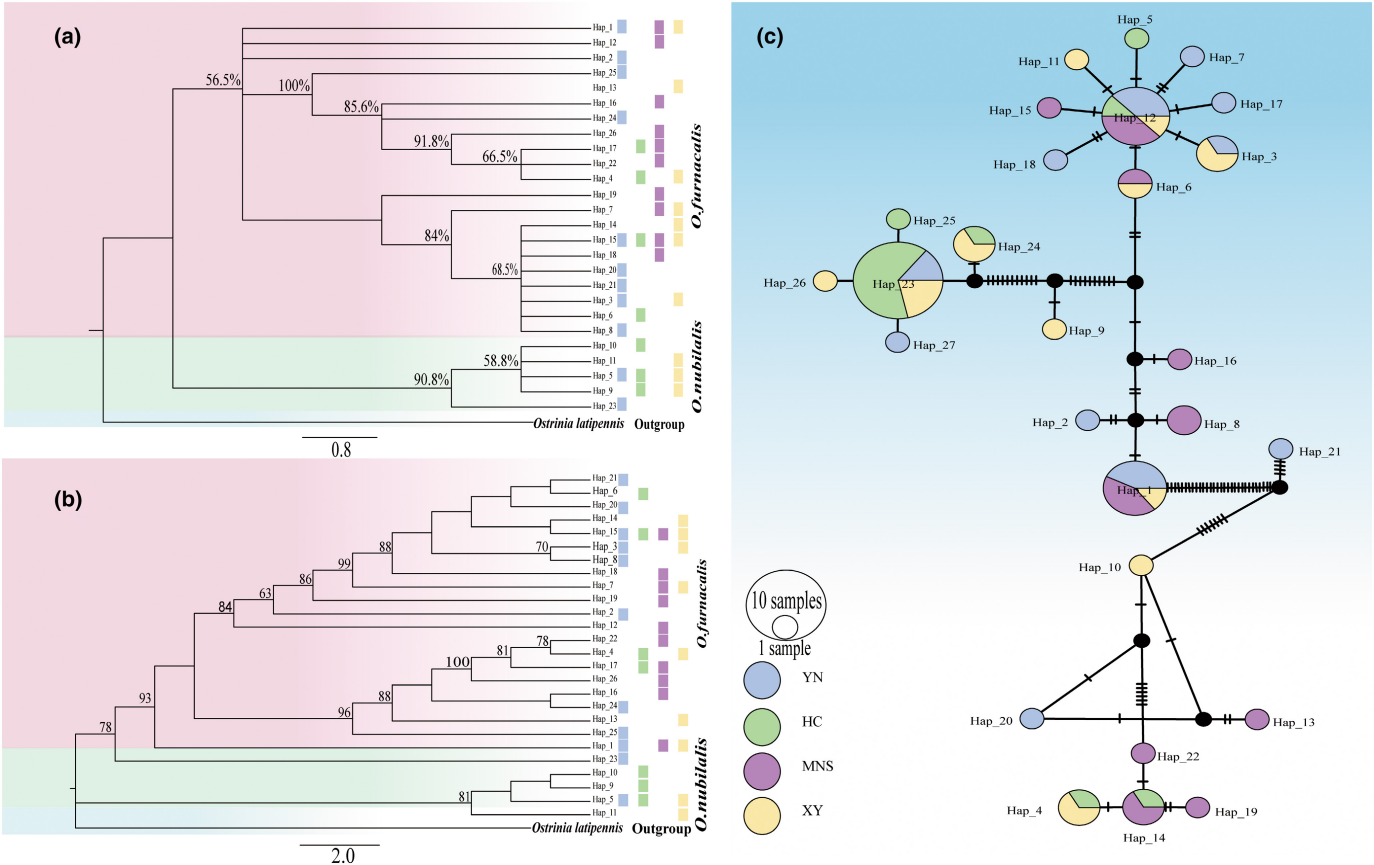
Phylogenetic trees (BI tree and ML tree) and TCS haplotype network. (a) BI tree from mtDNA haplotype dataset (MTHD). Numerals at nodes indicate Bayesian posterior probabilities (>50% are shown). (b) ML tree from mtDNA haplotype dataset (MTHD). Numerals at nodes indicate bootstrap values (>50 are shown). (c) TCS haplotype network constructed from mtDNA haplotype dataset (MTHD). The size of each circle is proportional to the frequency of the haplotypes. Black solid circles represent haplotypes either unsampled or extinct, and dashes represent the corresponding mutational steps.

We identified 27 haplotypes based on MTHD (Figure 2c). A total of 19 haplotypes (70.37% of all haplotypes) were unique to the four populations (YN, XY, HC, MNS). The H12 was the most common haplotype, shared by four populations. The haplotype H1 was shared by three populations (YN, XY, MNS), H23 was shared by three populations (YN, XY, HC), H3 by two populations (YN, XY), H4 by two populations (HC, XY), H6 by two populations (MNS, XY), and H14 by two populations (HC, MNS). However, there was no strong correspondence between haplotypes and their geographical distributions (Figure 2c).

DAMBE analysis showed that haplotype sequences were far from saturated and could be used for subsequent analyses (*p* < .5, Iss (0.0301) ≪ (0.7851)). For the MTHD dataset, phylogenetic analyses inferred with BI and ML methods were slightly different (Figure 2a,b). The topology inferred by the BI method showed that ECB haplotypes were grouped together in a distinct clade and clearly separated from the ACB clade, while the ML analysis showed that most ECB haplotypes (Hap10, Hap9, Hap5, Hap11) were grouped together except for the Hap23, which was nested into the ACB clade. In addition, haplotypes from the same locality were not lumped together supporting no geographical structuring (Figure 2). In contrast, phylogenetic analyses inferred with ML and BI methods based on NUHD showed that ACB and ECB were not recovered as monophyletic lineages (Appendix S7). The discordant results between MTHD and NUHD suggested that incomplete lineage sorting might be present between these two species.

### 3.3 Historical demography analyses

**FIGURE 3:**
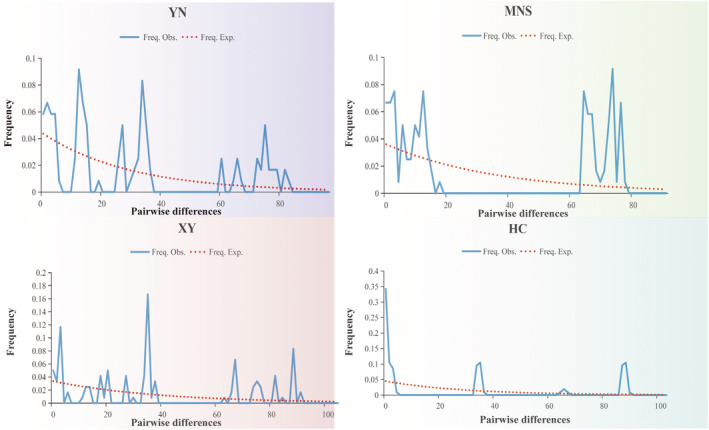
Mismatch distribution of four populations. *X* axis: Pairwise differences. *Y* axis: Frequency. Obs means the observed distribution of pairwise difference. Exp means the expected equilibrium distributions.

Neutral tests and mismatch distribution analysis were performed to detect possible range expansion events of the two species (ACB and ECB) and four geographical populations (YN, XY, HC, MNS). For the total sampling individuals of ACB, Tajima's *D* and Fu's *F*_s_ (1.30977 and 2.156) values were all positive based on MTD, while Tajima's *D* and Fu's *F*_s_ values (−0.90279 and −55.649) were all negative based on NUD. For ECB, Tajima's *D* and Fu's *F*_s_ values were −1.42151/−0.55939 and −1.282/−9.594 based on MTD and NUD, respectively (Appendix S4). In general, neutral test analysis of populations for each species based on MTD and NUD did not reach a significant level and were consistent with the “neutral evolution” hypothesis. Mismatch distribution analyses of four populations (YN, XY, HC, MNS) showed a multimodal (Figure 3), suggesting that the overall population size remains stable and no expansion events occurred in the recent past.

### 3.4 Divergence time analyses

**FIGURE 4:**
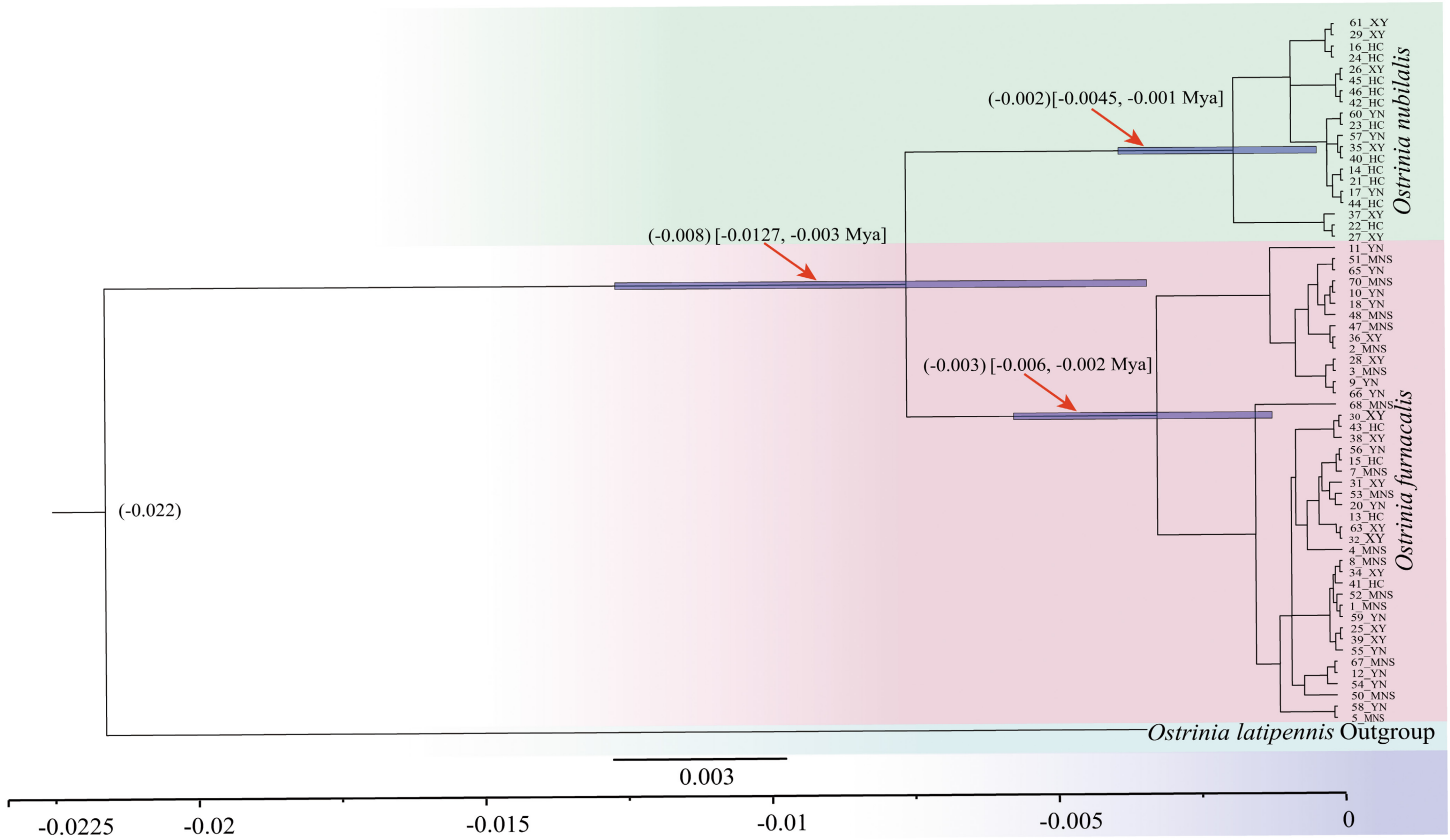
Chronogram of the ACB and ECB lineages derived from maximum clade credibility tree. The numeral at each node indicates the common ancestor of divergence time (Mya) with a 95% confidence interval. The numbers before sampling sites (YN, XY, HC and MNS) represent the voucher specimenID.

The BEAST analyses suggested that the first divergence time between the two species of ACB and ECB was estimated at 0.008 Mya (95% highest posterior density, HPD: 0.003–0.0127 Mya), the age of most recent common ancestor (MRCA) of ACB individuals was 0.003 Mya (95% HPD: 0.002–0.006 Mya), and the age of MRCA of ECB individuals was 0.002 Mya (95% HPD: 0.001–0.0045 Mya; Figure 4). In addition, the divergence time between the MRCA of ACB and ECB with the *O. latipennis* was estimated at 0.022 Mya (Figure 4). Our findings suggested that the population differentiation of the ACB and ECB most likely occurred in Holocene periods.

### 3.5 Gene flow analyses

The *N*_m_ was applied to identify the gene flow frequency within and between the two species across their different geographical populations (YN, XY, HC, MNS) based on MTD and NUD (Table 1). At the species level, the *N*_m_ values between the ACB and ECB ranged from 0.125 to 0.18 and from 0.25 to 1.054 based on MTD and NUD, respectively. For the three sympatric regions (YN, XY, HC), the values of *N*_m_ between the ACB and ECB ranged from 0.13 to 0.18 and from 0.25 to 0.575 based on MTD and NUD, respectively. For ACB, the *N*_m_ values between the different populations ranged from −97.15 to 2.17 and from −20.31 to 13.79 based on MTD and NUD, respectively (Table 1). For ECB, the *N*_m_ values between the different populations ranged from 0 to 23.49 and from −40 to 4.17 based on MTD and NUD, respectively. These results indicated that the levels of gene flow between the two species (ACB and ECB) based on MTD were low; however, gene flow among different geographical populations was high for both ACB and ECB.

**FIGURE 5:**
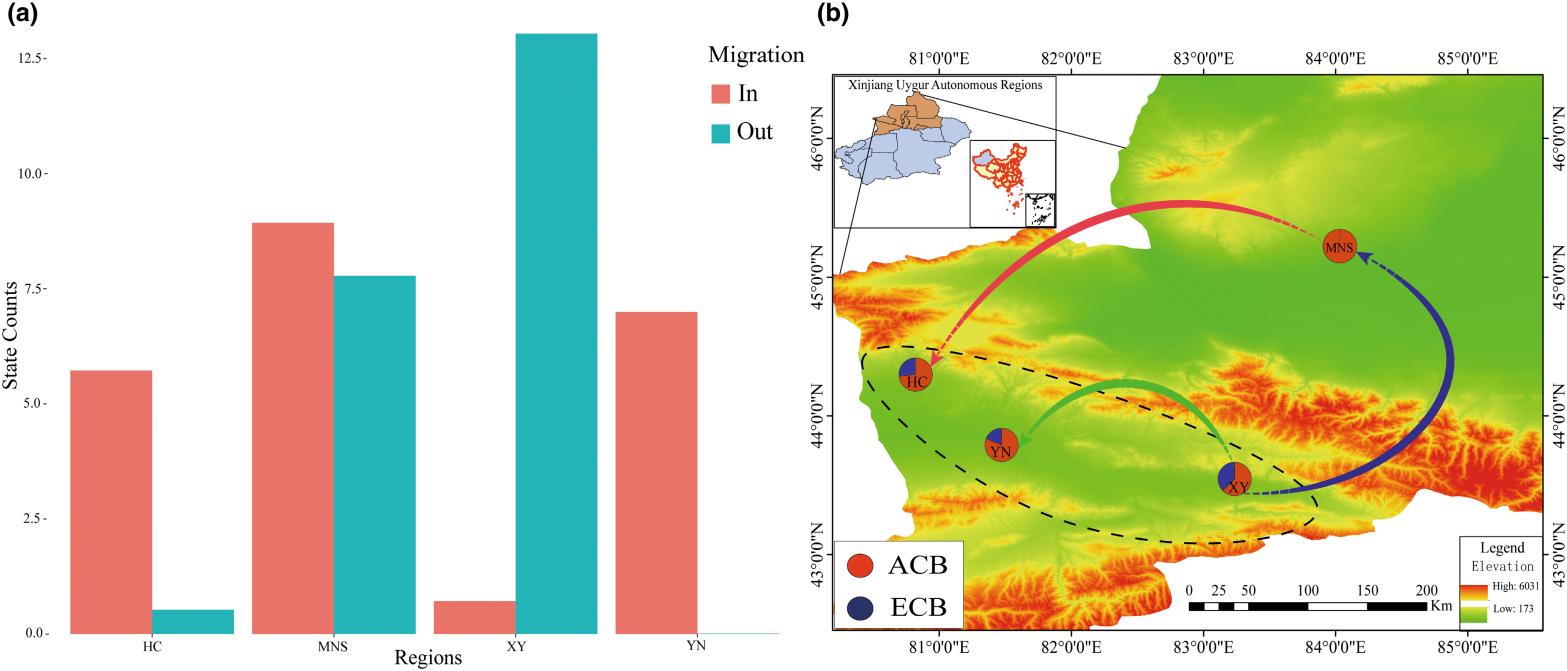
(a) Gene flow pathways and histograms of total number of state transitions for two lineages. The *X*‐axis of the histogram shows different regions, the *Y*‐axis shows the size of the migration. (b) Significant migratory pathways from one location to another are indicated on the maps.

BSSVS analyses (Table 2, Figure 5) showed that the occurrence of migration and diffusion in four populations (HC, YN, XY, MNS) was significantly different from each other. HC and YN had a large number of inflow migratory pathways. In contrast, XY had a large number of outflow migratory pathways, whereas MNS had almost equal inflows and outflows. In general, we found that population migratory route direction between different populations was outflow in XY and inflow in YN, HC (Figure 5). In addition, the statistics of BF and Indicator based on MTD were summarized in Table 2. Our results suggested that three potential gene flow pathways (BF > 3, Indicator > 0.5), namely from XY to MNS (BF/Indicator = 74.730/0.971), from XY to YN (12.171/0.844), and from MNS to HC (372.669/0.994), were observed between the pair two populations.

**TABLE 2:** BAYES_FACTOR (BF) and indicator based on mitochondrial genes dataset (MTD).

From	To	BAYES_FACTOR(BF)	Indicator
HC	YN	1.588	0.412
HC	MNS	0.751	0.250
HC	XY	0.611	0.214
YN	MNS	1.872	0.454
YN	XY	1.074	0.323
MNS	XY	0.873	0.280
YN	HC	1.283	0.363
MNS	HC	372.669	0.994
XY	HC	0.522	0.188
MNS	YN	2.675	0.543
XY	YN	12.171	0.844
XY	MNS	74.730	0.971

## 4 DISCUSSION

### 4.1 Genetic differentiation and demographic history of ACB and ECB in sympatric regions within the Yili area

Our findings indicate that there is high genetic differentiation between the two species (ACB and ECB) in Xinjiang. However, the degree of genetic differentiation among different geographical populations within each species is low. These results are consistent with previous studies reported that a low level of genetic differentiation is present among Chinese populations of ACB (Bourguet et al., 2014; Li et al., 2014). Furthermore, our molecular variance analysis (AMOVA) indicates that a large proportion of the total genetic variance is attributed to variations within populations (Appendix S5). Moreover, genetic distance and Mantel test results show that there is no significant correlation between geographical distance and genetic distance, suggesting that genetic variation among populations of corn borers may not be associated with geographical distance but most likely related to biological characteristics and host plants differentiations among populations proposed by (Li et al., 2014; Wang et al., 2018). By contrast, Coates et al. (2011) and Kim et al. (2011) suggested that significant genetic differentiation may present among intermountain regions due to topographic barriers in the Eastern United States based on microsatellite and SNP markers.

Our neutrality tests results (Tajima's *D* and Fu's *F*_s_, shown in Appendix S4 and Figure 3) indicate that recent population expansion has not occurred within each of four populations (Fu, 1997; Harpending et al., 1998; Tajima, 1989). In addition, the mismatch distribution among haplotypes is multimodal, further supporting there being no population expansion within these populations. However, limitation of markers used in the present study and small sample size may restrict to illuminate the fine‐scale population structure and genetic variation in Xinjiang for both ACB and ECB. Further investigations based on additional markers and comprehensive sampling from different populations in the Yili valley and surrounding areas are essential to reveal their genetic differentiation patterns and demographic history in Xinjiang.

### 4.2 Gene flow and migratory pathways between the different populations of ACB and ECB

According to our gene flow analysis (Table 1), our results show that a high level of gene flow exists between populations for both ACB and ECB, which corresponds to the conclusion that a low degree of genetic differentiation is present among populations within ACB and ECB due to their strong ability to spread (Li et al., 2010, 2014; Yang et al., 2015). In addition, a low degree of gene flow was found between ACB and ECB within the sympatric Yili area in Xinjiang, which is consistent with the gene flow estimates based on concatenated *COI*‐*COII* mitochondrial haplotypes between locations where ACB and ECB co‐occur (Wang et al., 2017). However, this is inconsistent with previous studies on other species pairs of the genus *Ostrinia* (Bourguet et al., 2014; Coates et al., 2013; Malausa et al., 2005). According to high‐throughput SNP and microsatellite markers and based on concatenated *COI* & *COII*, Wang et al. (2017) confirmed that the Yili area is a hybrid zone between ACB and ECB and found that there is gene flow from the invading ACB into ECB. Therefore, we hypothesize that reinforcement might be the major driver of reproduction isolation of ACB and ECB in sympatry. However, the molecular mechanisms of hybrids and the possible causes of the reinforcement and introgressive hybridization of ACB and ECB in sympatry are unclear yet, and further investigation based on genomic data, which provides greater inferential resolution for resolving fine‐scale phylogeographic patterns, is needed.

According to migratory pathways between the different populations of ACB and ECB, three main gene flow pathways are observed, namely XY to MNS, XY to YN, and MNS to YN. In the present study, MNS is located in the north of the three regions (YN, HC and XY), not in the Yili River Valley where the overall topography of the middle vein of the Tianshan Mountains opens westward. However, gene flows were found between MNS of ACB and the other three populations of ACB and ECB in the Yili River Valley, suggesting that the Tianshan Mountains might have many refuges according to the “glacial refugia” hypothesis (Avise, 1992; Smith, 1965). In this scenario, the Tianshan Mountains are less likely a barrier to gene flow between ACB and ECB based on the evidence observed in the present study. However, further investigation on more samples of ACB and ECB from both sides is needed.

### 4.3 Evolution pattern of ACB and ECB in sympatric regions within the Yili area

Given the divergence time between ACB and ECB is approximately 0.008 Mya (0.003–0.0127 Mya), this implies that the interspecific divergence occurred in Holocene in the Yili area. Meanwhile, the second intraspecific divergence time of ACB was estimated at 0.003 Mya (0.002–0.006 Mya) and observed at 0.002 Mya (0.0045–0.001) for ECB. Our findings indicate that large‐scale climate changes associated with tectonic events during early Holocene may have caused considerable fragmentation of habitats for sympatric separation of ACB and ECB in the Yili area (Médail & Diadema, 2009), and various geographical topography (e.g., basins, rivers, canyons) presumably promoted the intraspecific divergence of ACB and ECB during the Holocene period. For example, the common ancestor of ACB and ECB in different glacial refugia moved to higher altitudes when the Holocene climate generally warmed, this may lead to fragmentation of habitats and genetic differentiation among isolated geographical populations (Bansal et al., 2013; Schluter, 2009; Zheng et al., 2002). In general, our results preliminarily reveal that the evolution pattern of ACB and ECB in this sympatric region is likely consistent with the Holocene climatic oscillations and “mountain isolation” hypothesis (Hewitt, 1996; Pyron & Burbrink, 2010). However, our proposed hypotheses should be tested through multiple lines of methods such as deep phylogeographic and population genetic structure investigation on ACB and ECB in this area.

## 5 CONCLUSION

Our results highlight that there is an asymmetrical gene flow pattern among four different geographical populations of ACB and ECB in the Tianshan Mountains where they are less likely a barrier to gene flow of the two species. The geological factors and climatic oscillations during the Holocene may have driven genetic patterns of two species. However, to fully understand the genetic variation and demographic history of corn borers in Xinjiang, and the gene flow between ACB and ECB in the sympatric Yili area, it will be necessary to expand the range of specimen collection and use additional genes in further investigation.

## AUTHOR CONTRIBUTIONS

**Bing Li:** Software (lead); writing – original draft (lead); writing – review and editing (lead). **Zhaofu Yang:** Project administration (lead).

## CONFLICT OF INTEREST

The authors declare no competing interest.

## Supporting information


Appendix S1

## Data Availability

The data that support the findings of this study are openly available in GenBank of NCBI as accession numbers (*COI*, MK286109‐MK286173; *COII*, MK286174‐MK286237; *Cytb*, MK286307‐MK286374; *RpS5*, MK286238‐MK286306; *CAD*, MK292148‐MK292216; *Wingless*, MK292217‐MK292285; *EF‐1α*, MK303832‐MK303897). The sequence datasets and appendixes in this study are available in Dryad Digital Repository (https://doi.org/10.5061/dryad.2547d7wsw).
