# Supplementary material for: Multilocus evidence provides insight into the demographic history and asymmetrical gene flow between Ostrinia furnacalis and Ostrinia nubilalis (Lepidoptera: Crambidae) in the Yili area, Xinjiang, China
Source: Ecol Evol. 2022 Nov 16;12(11):e9504. doi: 10.1002/ece3.9504 (PMC9667411; doi:10.1002/ece3.9504)
Supplement: Supplementary file 1 — Appendix S1 [file ECE3-12-e9504-s001.docx]

**Appendix information**

**Appendix S1**. Sample information of different geographical populations of *Ostrinia furnacalis* (ACB) and *Ostrinia nubilalis* (ECB) used in this study.

| **Ecotype** | **Locality** | **Sample size** | **ACB Sample size** | **ECB Sample size** | **Collection dates** |
| --- | --- | --- | --- | --- | --- |
| Sympatric area in Kazk | Yining (YN) | 18 | 12 | 6 | 31.07.2017-02.08.2017 |
| Sympatric area in Yili River Valley, Xinjiang, China | Xinyuan (XY)  Huocheng (HC) | 18  15 | 12  11 | 6  4 | 27.07.2017-28.07.2017  04.08.2017-05.08.2017 |
| Allopatric area in the North of Xinjiang, China | Manasi (MNS) | 19 | 19 | - | 07.08.2017-11.08.2017 |

**Appendix S2**. PCR primers used in this study.

| **Gene region** | **Name** | **Sequence (forward and reverse) (5’-3’)** | **Size (bp)** | **References** |
| --- | --- | --- | --- | --- |
| *COI* | LCO1490  HCO2198 | GGTCAACAAATCATAAAGATATTGG  TAAACTTCAGGGTGACCAAAAAATCA | 710 | Folmer *et al*. (1994) |
| *COII* | TL2J3037  TKN3785 | ATGGCAGATTAGTGCAATGG  GTTTAAGAGACCAGTACTTG | 739 | Simon *et al* (1994)  Wang & Long (2003) |
| *Cytb* | CB1  CB2 | TATGTACTACCATGAGGACAATATC  ATTACACCTCCTAATTTATTAGGAAT | 429 | Simon *et al.* (1994) |
| *Wingless* | LepWg2  LepWg1 | ACTICGCRCACCARTGGAATGTRCA  GARTGYAARTGYCAYGGYATGTCTGG | 392 | Brower & DeSalle (1998) |
| *EF-1α* | ELF2F (f)  efrcM4 (r) | AAAATGCCCTGGTTCAAGGGA  ACAGCVACKGTYTGYCTCATRTC | 642 | Wan *et al*. (2013)  Monteiro & Pierce (2001) |
| *RpS5* | RpS5r  RpS5f | CGGTTRGAYTTRGCAACACG  ATGGCNGARGARAAYTGGAAYGA | 597 | Haines & Rubinoff (2012)  Wahlberg & Wheat (2008) |
| *CAD* | CAD4_Pyr_R  CAD4_Pyr_F | CKRTCACTCATGTCRTA  GAAGAAGCATTTCAAAAAGC | 615 | Haines & Rubinoff (2012) |

Brower, A.V. & Desalle, R. (1998) Patterns of mitochondrial versus nuclear DNA sequence divergence among nymphalid butterflies: the utility of wingless as a source of characters of phylogenetic inference. *Insect Molecular Biology, 7,* 73–82

Folmer, O., Black, M., Hoeh, W., Lutz, R. & Vrijenhoek, R. (1994) DNA primers for amplification of mitochondrial cytochrome coxidase subunit I from diverse metazoan invertebrates. *Molecular Marine Biology and Biotechnology,* *3,* 294–299.

Haines, W. P. & Rubinoff, D. (2012) Molecular phylogenetics of the moth genus *Omiodes Guenée* (Crambidae:Spilomelinae), and the origins of the Hawaiian lineage. *Molecular Phylogenetics and Evolution, 65,* 305–316.

Monteiro, A. & Pierce, N.E. (2001) Phylogeny of *Bicyclus* (Lepidoptera: Nymphalidae) inferred from *COI*, *COII*, and *EF-1α* Gene Sequences. *Molecular Phylogenetics* *and Evolution,* *18,* 264–281.

Simon, C., Frati, F., Beckenbach, A., Crespi, B., Liu, H. & Flook, P. (1994) Evolution, weighting, and phylogenetic utility of mitochondrial gene sequences and a compilation of conserved polymerase chain reaction primers. *Annals of the Entomological Society of America,* *87,* 651–701.

Wan, J., Kim, M.J., Cho, Y., Jun, J., Jeong, H.C., Lee, K.Y. & Kim, I. (2013) Sequence divergence and phylogenetic investigation of the *Nymphalidae* (Lepidoptera: Papilionoidea) occurring in South Korea. *International Journal of Industrial Entomology,* *26,* 95–112.

Wahlberg, N. & Wheat, C.W. (2008) Genomic outposts serve the phylogenomic pioneers: designing novel nuclear markers for genomic DNA extractions of Lepidoptera. *Systematic Biology, 57,* 231–242.

Wang, R. & Long, Y. (2003) An improved COII primer specific for Lepidoptera. *Biochemical Genetics, 41,* 57–59.

**Appendix S3**. Variation of individual genes, mitochondrial genes dataset (MTD) and nuclear genes dataset (NUD).

| **Gene** | **N** | **Length of sequence (bp)** | **C** | **V** | **Pi** | **S** | **Ts** | **Tv** | **R** | **A + T**  **(%)** |
| --- | --- | --- | --- | --- | --- | --- | --- | --- | --- | --- |
| *COI* | 65 | 655 | 636 | 19 | 15 | 4 | 5 | 0 | 12.2 | 70.4 |
| *COII* | 64 | 739 | 724 | 15 | 11 | 4 | 4 | 0 | NC | 77.0 |
| *Cytb* | 68 | 429 | 371 | 58 | 51 | 7 | 10 | 9 | 1.2 | 74.9 |
| *CAD* | 69 | 599 | 567 | 32 | 17 | 15 | 2 | 1 | 1.7 | 62.5 |
| *EF-1α* | 66 | 642 | 627 | 15 | 8 | 7 | 2 | 1 | 2.9 | 42.6 |
| *RPS5* | 69 | 610 | 599 | 11 | 7 | 4 | 2 | 0 | 4.2 | 49.4 |
| *Wingless* | 69 | 392 | 369 | 23 | 14 | 9 | 4 | 0 | 12.3 | 29.8 |
| *COI + COII + Cytb* | 63 | 1823 | 1731 | 92 | 76 | 16 | 19 | 8 | 2.4 | 74.1 |
| *CAD* + *EF-1α* + *RPS5* + *Wingless* | 66 | 2243 | 2163 | 80 | 46 | 34 | 9 | 2 | 3.8 | 47.5 |

N: the number of sequences; C: conserved sites; V: variable sites; Pi: parsimony-informative sites; S: singleton sites; Ts: transition; Tv: transversion; R: transition / transversion; A+T (%): A+T content.

**Appendix** **S4.** The genetic diversity and neutrality tests based on mitochondrial genes dataset (MTD) and nuclear genes dataset (NUD) for *Ostrinia furnacalis* (ACB) and *Ostrinia nubilalis* (ECB) and four geographical populations.

| **Species** | **Number of samples** | **Number of haplotypes** | **Haplotype diversity**  **（*Hd* ± SD）** | **Nucleotide diversity**  **（𝜋 ± SD）** | **Tajima’s *D*** | **Fu’s *Fs*** |
| --- | --- | --- | --- | --- | --- | --- |
| ***O. furnacalis*** | 43 / 48 | 22 / 48 | 0.934 ± 0.022 /  1 ± 0.004 | 0.01366 ± 0.00164 /  0.0041 ± 0.00019 | 1.30977 /  -0.90279 | 2.156 /  -55.649 |
| **XY** | 10 / 12 | 8 / 12 | 0.956 ± 0.059 /  1 ± 0.034 | 0.01552±0.00158 /  0.00347±0.00078 | 1.04567 /  -0.71754 | 1.932 /  -5.977 |
| **YN** | 13 / 15 | 9 / 15 | 0.923 ± 0.057 /  1 ± 0.024 | 0.01046±0.00146 /  0.00400±0.00075 | -0.58466 /  -0.11627 | 1.896 /  -8.062 |
| **HC** | 4 / 4 | 4 / 4 | 1.000±0.031 /  1±0.177 | 0.01810±0.00212 /  0.00401±0.00097 | 2.18573 /  0.31839 | 1.650 /  0.210 |
| **MNS** | 16 / 17 | 10 / 17 | 0.933 ± 0.040 /  1 ± 0.02 | 0.01466 ± 0.00252 /  0.00451 ± 0.00039 | 1.93648 /  -0.31121 | 3.693 /  -9.237 |
| ***O. nubilalis*** | 20 /18 | 5 / 18 | 0.505 ± 0.126 /  1 ± 0.019 | 0.00051 ± 0.00015 / 0.00492 ± 0.00048 | -1.42151 /  -0.55939 | -1.282 /  -9.594 |
| **XY** | 6/5 | 3/5 | 0.733 ± 0.155 /  1 ± 0.126 | 0.00095 ± 0.00048 /  0.00535±0.00103 | -0.05722 /  0.64567 | 0.758 /  -0.101 |
| **YN** | 3/2 | 2/2 | 0.667 ± 0.097 /  1 ± 0.500 | 0.00037 ± 0.00037 /  0.00357 ± 0.00126 | - / - | - / - |
| **HC** | 11/11 | 3/11 | 0.345 ± 0.172 /  1.000 ± 0.039 | 0.00030 ± 0.00032 /  0.00503 ± 0.00095 | -1.59996 /  -0.71861 | -0.537 /  -.801 |

Values reported as follow: MTD / NUD. “-” indicated that values of *Tajima’s D* and *Fu’s. Fs* were not observed.

**Appendix S5**. Analysis of molecular variance (AMOVA) results for mitochondrial genes dataset (MTD) and nuclear genes dataset (NUD).

| **Genes** | **Source of variation** | ***df*** | **Sum of squares** | **Variance**  **components** | **Percentage of variation** |
| --- | --- | --- | --- | --- | --- |
| *COI* + *COII* + *Cytb* | Among groups | 1 | 303.982 | 1. 90967 Va | 55.83 |
|  | Among populations within groups | 5 | 32.434 | -0. 28877 Vb | -1.48 |
|  | Within populations | 56 | 499.489 | 8.91944 Vc | 45.65 |
|  | Total | 62 | 835.905 | 19.54034 | 100 |
| *CAD* + *EF-1α* + *RpS5* + *Wingless* | Among groups | 1 | 48.782 | 1.66848 Va | 25.60 |
|  | Among populations within groups | 5 | 25.326 | 0.02798 Vb | 0.43 |
|  | Within populations | 59 | 284.438 | 4.82099 Vc | 73.97 |
|  | Total | 65 | 358.545 | 6.51745 | 100 |

Significance test: 1000 permutations; *df*: degrees of freedom.

**Appendix S6**. The natural logarithm of geographical distance (km) (below the diagonal) and pairwise genetic distance (above the diagonal) between different geographical populations based on mitochondrial genes dataset (MTD) and nuclear genes dataset (NUD). The natural logarithm was used to reduce of absolute values of geographical distance (km) and avoid the heterogeneity in Mantel test.

|  | **YN** | **XY** | **HC** | **MNS** |
| --- | --- | --- | --- | --- |
| **YN** |  | 0.014 / 0.005 | 0.015 / 0.006 | 0.014 / 0.004 |
| **XY** | 5.025 |  | 0.014 / 0.005 | 0.017 / 0.005 |
| **HC** | 3.969 | 5.318 |  | 0.019 / 0.006 |
| **MNS** | 5.930 | 5.546 | 6.057 |  |

Values reported as follow: MTD / NUD.

**Appendix S7.** Phylogenetic trees (BI tree and ML tree). (a) BI tree from nuDNA haplotype dataset (NUHD). Numerals at nodes indicate Bayesian posterior probabilities (>50% are shown). (b) ML tree from nuDNA haplotype dataset (NUHD). Numerals at nodes indicate bootstrap values (>50 are shown). Pink clades indicate *Ostrinia furnacalis* (ACB), green clades indicate *Ostrinia nubilalis* (ECB).

**
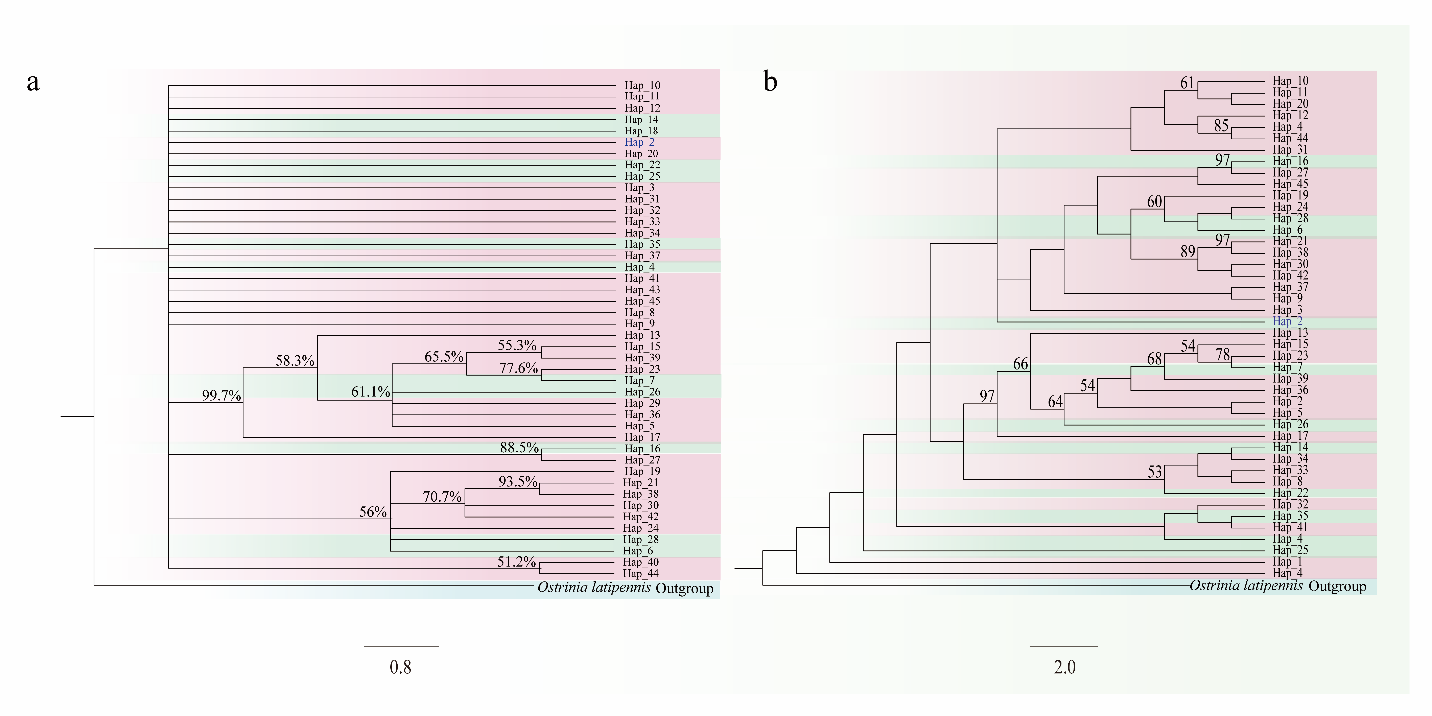
**
